# Anti-factor H Autoantibody-Associated Hemolytic Uremic Syndrome: A Rare Entity in a Pediatric Patient

**DOI:** 10.7759/cureus.60872

**Published:** 2024-05-22

**Authors:** Gaurav Singh, Pavan Wakhare, Atul D Sajgure, Charan Bale, Nilesh Shinde

**Affiliations:** 1 Nephrology, Dr. D. Y. Patil Medical College, Hospital and Research Centre, Pune, IND

**Keywords:** atypical hemolytic uremic syndrome, anti-factor h antibody, thrombotic thrombocytopenic purpura, ahus, complement mediated hus, thrombotic microangiopathies

## Abstract

An 11-year-old patient presented with the primary complaint of hematuria and vomiting. On further investigation and a series of diagnostic tests, including a biopsy and thrombotic microangiopathy (TMA) profile, the patient was diagnosed with thrombotic microangiopathy. TMA is a pathological process involving endothelial cell injury, leading to thrombocytopenia and microangiopathic hemolytic anemia. This case highlights the importance of considering TMA in pediatric patients presenting with nonspecific symptoms, such as loss of appetite. Further research is needed to understand the pathophysiology and optimal management strategies for pediatric TMA. This case adds to the growing body of literature on pediatric TMA and underscores the need for a high index of suspicion in similar clinical scenarios.

## Introduction

Anti-factor H antibody-associated hemolytic uremic syndrome (AFH-HUS) is a rare, life-threatening disease characterized by the triad of microangiopathic hemolytic anemia, thrombocytopenia, and acute kidney injury. It is a subtype of HUS that is caused by autoantibodies against complement factor H (CFH), a key regulator of the alternative pathway of the complement system. The proposed mechanism for the development of HUS is a trigger event, such as infection or pregnancy, in a susceptible individual with a gene variant(s) or antibodies to complement proteins, which leads to uninhibited continuous activation of the alternative pathway resulting in the formation of the membrane attack complex. This causes kidney endothelium damage leading to activation of the coagulation cascade and thrombotic microangiopathy [1]. Complement factor H (CFH) antibodies of the immunoglobulin (Ig) G (IgG) class have been reported in approximately 8-10% of patients with atypical HUS [2]. This study enlightens the importance that pediatric patients may have HUS associated with anti-factor H autoantibody, which is an autoimmune complement-mediated hemolytic uremic syndrome (autoimmune CM-HUS). While most of the pediatric age group patients present with diarrhea-associated HUS, here we discuss a case of an 11-year-old female who presented with hematuria and vomiting and was then extensively worked up for all possible causes of hemolysis which included thrombotic microangiopathy (TMA) panel and unexpectedly anti-factor H autoantibody came positive. AFH-HUS children have the risk of progression from acute kidney injury (AKI) to chronic kidney disease (CKD). However, appropriate therapy with plasma exchange and immunosuppression can help in reducing the risk of progression, thereby making the kidney injury reversible [3].

## Case presentation

An 11-year-old female presented to the emergency department with complaints of blood in urine and vomiting, which was sudden in onset. The patient was then evaluated and was found to have hypertension, acute kidney injury, and hemolysis. She had no history of any other comorbidities. No history of any drug abuse.

On examination, she was conscious and oriented but had a blood pressure of 180/110 mmHg, pulse of 120 beats per min, respiratory rate of 18 breaths per min, and temperature of 99.2°F. On auscultation of the chest, she had bilateral air entry with bilateral crepitations. On precordial examination, she had normal heart sounds. There were no murmurs. Abdominal examination was unremarkable. Neurological examination showed no neurological deficit. Electrocardiogram showed sinus tachycardia. Ultrasonography of the abdomen pelvis was done which showed normal-sized kidneys (right kidney 108 mm, left kidney 109 mm) with raised cortical echogenicity. In view of severely deranged creatinine and urea, hemodialysis was initiated. Laboratory investigations showed elevated urea and creatinine. Table 1 shows laboratory investigations over the course of her stay in hospital and on follow-up.

**Table 1:** Laboratory investigation trend of over 27 days. PCR: protein creatinine ratio; ESR: erythrocyte sedimentation rate; LDH: lactate dehydrogenase; FBS: fasting blood sugar; Sr: serum

Investigations	Day 1	Day 3	Day 5	Day 7	Day 9	Day 11	Day 21	Day 27	Reference range
Hemoglobin	6.8	7.2	6.6	10.8	10.3	10.1	7.7	9.5	12.0-14.5 g/dL
Total leucocyte count	9090	10280	14320	16220	13650	12750	10900	12710	4000-10800/µL
Platelets	36000	56000	80000	116000	141000	175000	89000	194000	150000-410000/µL
Sr creatinine	4.6	6.31	-	-	5.1	5.30	1.15	1.04	0.59-1.04 mg/dL
Urea	169	174	-	-	-	119	50	25	17-49 mg/dL
Sr sodium	138	134	132	134	-	132	141	136	136-145 mmol/L
Sr potassium	4.7	4.0	5.1	4.2	-	4.6	3.56	3.6	3.50-5.10 mmol/L
ESR	-	104	-	-	-	-	-	-	<25 mm
Total protein	-	6.2	-	-	-	-	-	-	6.4 to.8.3 g/dL
Urine PCR	-	32.5	-	-	-	-	-	6.31	<0.2
Retic count	7.25%	8.99%	-	-	-	-	-	-	0.5-2.5%
LDH	-	1863	1566	846	772	559	-	-	110-283 U/L
Ca	-	-	7.5	-	-	7.6	-	-	8.6-10.2 mg/dL
FBS	156	120	131	90	126	99	-	-	<100 mg/dL
Mg	-	-	-	-	-	-	-	-	1.80-2.40 mg/dL

Peripheral smear showed macrocytosis, anisocytosis, polychromasia, schistocytes along with spherocytes. Direct Coomb’s test was also done which came out to be negative. Urine routine at the time of presentation showed massive proteinuria (Table 2). To ascertain whether autoimmune and complement-mediated diseases were a factor, we conducted C3 and C4 tests. Unexpectedly, C3 came out to be low (Table 3).

**Table 2:** Urine routine/microscopy findings of the patient.

Urine routine/microscopy	Patient values	Reference range
Urine albumin	++++	Negative
Urine RBC	80-100 RBC/hpf	0-2 RBC/hpf
Urine WBC	15-20 WBC/hpf	<5 WBC/hpf
Urine epithelial cells	15-20	0-2
Casts	Absent	Absent

**Table 3:** C3 and C4 complement levels.

Lab investigation	Patient values	Reference range
C3	85.75 mg/dL	90-180 mg/dL
C4	15.53 mg/dL	9-36 mg/dL

Hemolysis along with thrombocytopenia was observed so thrombotic microangiopathy (TMA) was suspected. The patient’s anti-factor H antibody was sent, which surprisingly came positive (patient's value 200 AU/mL, normal range 0-150 AU/mL). Other parameters of TMA panel like ADAMTS13, AH50, CH50, and C3 nephritic factor came negative. Hence, autoimmune complement-mediated HUS (autoimmune CM-HUS) was diagnosed. The decision was made to proceed with plasmapheresis, and the process was subsequently started. Renal biopsy was done to identify the extent of ischemic injury sustained as a result of a renal TMA in order to know the degree of irreversible kidney injury.

**Figure 1:**
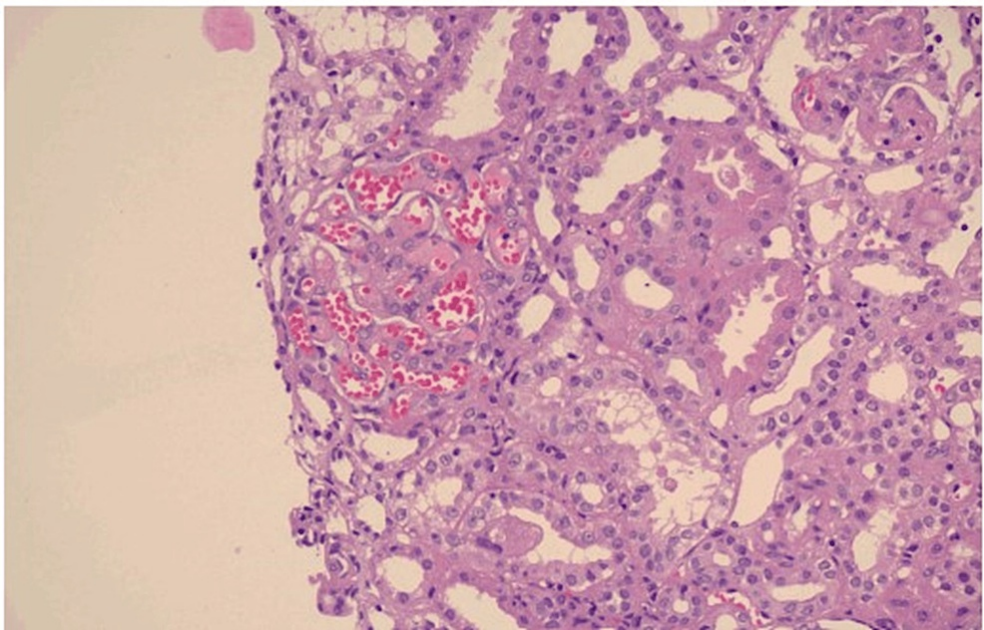
Light microscopy of renal biopsy specimen at 20×10 magnification showing subendothelial edema, ectatic capillary loops, focal mesangiolysis, and RBC fragmentation with capillary tuft retraction.

Once renal biopsy was done we found that on light microscopy, the glomeruli showed subendothelial edema, ectatic capillary loops, focal mesangiolysis, and RBC fragmentation with capillary tuft retraction. Few glomeruli show capillary wall double contours. There was no evidence of crescent formation/proliferative activity/tuft necrosis in the visualized glomeruli. Tubular atrophy and interstitial fibrosis involve <10% of the sampled cortex. The tubules show acute injury with few granular casts in tubular lumina. Interstitial inflammation is minimal. Arteries and arterioles showed subintimal edema. Segmental necrosis and luminal thrombotic occlusion of a few arterioles including hilar branches are noted (thrombotic microangiopathy) (Figure 1).

**Figure 2:**
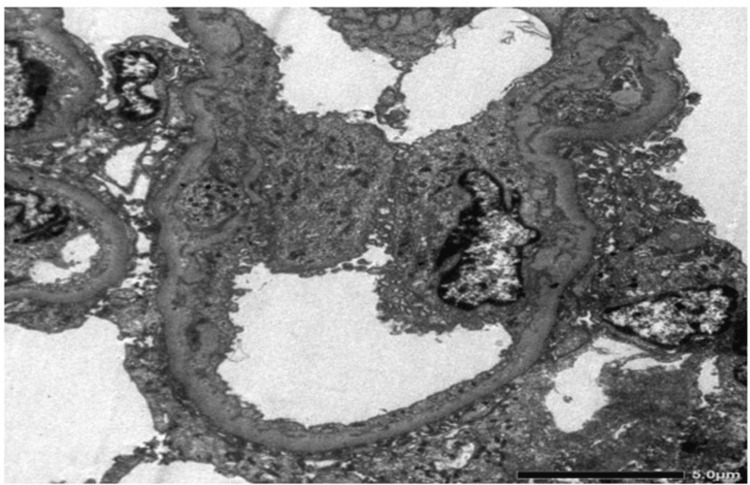
Electron microscopy of renal biopsy specimen showing foot process flattening with vacuolization of the lamina densa.

On Immunofluorescence, C3 came positive with 1+ mesangial granular pattern. IgA, IgG, IgM, and C1q came negative. Kappa and lambda light chains also came negative. Electron microscopy revealed mesangial widening with a few regions showing three to four cells per mesangial region. Ninety percent of the loops reveal foot process flattening. Some loops show wrinkling and occasional loops reveal vacuolization of the lamina densa. Most of the loops show loss of fenestrae of the endothelial cells. Few endothelial cells are prominent in areas which is consistent with thrombotic microangiopathy (Figure 2).

Six cycles of plasmapheresis were done on days 5, 7, 9, 11, 13, and 15 along with pulse methylprednisolone therapy. Adequate hydration was maintained along with other supportive treatments. A rise in platelets was observed just after the first session of plasmapheresis and so with the serum lactate dehydrogenase (LDH) levels. Whereas a fall in serum creatinine was observed after the fourth cycle of plasmapheresis. Her urine output improved over the course of days. Prophylactically antibiotics were also started and platelet transfusion were given.

On the 21st day, the patient was discharged with the following laboratory values: urea at 50 mg/dL, serum creatinine at 1.15 mg/dL, serum sodium at 141 mEq/dL, serum potassium at 3.56 mEq/dL, and platelets at 89000/mm^3^. Upon follow-up after six days, the laboratory results showed a urea 25 mg/dL and serum creatinine of 1.04 mg/dL. The patient was fully recovered with prompt treatment and other supportive management.

## Discussion

Anti-factor H antibody-associated hemolytic uremic syndrome, also known as aHUS, is an uncommon and potentially fatal condition. It is marked by the following three main features: microangiopathic hemolytic anemia, thrombocytopenia, and acute kidney damage. This particular form of HUS is triggered by autoantibodies that target complement factor H (CFH), a crucial component in regulating the alternative pathway of the complement system [1]. In the pediatric population, aHUS presents a unique challenge due to its rarity and the severity of its symptoms. Early recognition and prompt treatment are crucial for improving patient outcomes [2]. Atypical hemolytic uremic syndrome (aHUS) is a rare form of thrombotic microangiopathy that associates, in 70% of cases, with genetic or acquired disorders leading to dysregulation of the alternative pathway of complement. Autoantibody directed against factor H causes at least 6-10% of aHUS cases. Activation of the alternative pathway of complement at the onset of disease portends a poor prognosis. Low C3 levels may occur in patients with factor H and factor I mutations causing anti-factor H production, just as seen in the present case [4].

## Conclusions

In conclusion, the presence of anti-factor H antibody plays a crucial role in pediatric patients presenting with features of thrombotic microangiopathy. This antibody is significant as it can lead to uncontrolled complement activation, resulting in microvascular thrombosis and organ damage. Early detection of this antibody can guide the diagnosis, allowing for prompt initiation of appropriate treatment. Timely intervention, including plasmapheresis and immunosuppressive therapy, can significantly improve the prognosis. Therefore, the importance of anti-factor H antibody cannot be overstated in the management of thrombotic microangiopathy in pediatric patients. Prompt treatment not only alleviates the symptoms but also aids in curing the illness, underscoring the need for early diagnosis and immediate therapeutic intervention.
